# Resveratrol increases *BRCA1* and *BRCA2* mRNA expression in breast tumour cell lines

**DOI:** 10.1038/sj.bjc.6600983

**Published:** 2003-07-01

**Authors:** P Fustier, L Le Corre, N Chalabi, C Vissac-Sabatier, Y Communal, Y-J Bignon, D J Bernard-Gallon

**Affiliations:** 1 Laboratoire d'Oncologie Moléculaire, Centre Jean Perrin, 58 Rue Montalembert, BP 392, 63011 Clermont-Ferrand, France; 2 Laboratoire d'Immunologie, Centre Jean Perrin, 58 Rue Montalembert, BP 392, 63011 Clermont-Ferrand, France

## Abstract

The phytochemical resveratrol, found in grapes, berries and peanuts, has been found to possess cancer chemopreventive effects by inhibiting diverse cellular events associated with tumour initiation, promotion and progression. Resveratrol is also a phyto-oestrogen, binds to and activates oestrogen receptors that regulate the transcription of oestrogen-responsive target genes such as the breast cancer susceptibility genes *BRCA1* and *BRCA2*. We investigated the effects of resveratrol on *BRCA1* and *BRCA2* expression in human breast cancer cell lines (MCF7, HBL 100 and MDA-MB 231) using quantitative real-time RT–PCR, and by perfusion chromatography of the proteins. All cell lines were treated with 30 *μ*M resveratrol. The expressions of *BRCA1* and *BRCA2* mRNAs were increased although no change in the expression of the proteins were found. These data indicate that resveratrol at 30 *μ*M can increase expression of genes involved in the aggressiveness of human breast tumour cell lines.

Resveratrol is a natural phytoalexin compound found in grapes and other food products. It has been found to possess oncopreventive activity by inhibiting ribonucleotide reductase (Fontecave *et al*, 1998) and cellular events associated with cell proliferation and tumour initiation, promotion and progression (Jang *et al*, 1997; Mgbonyebi *et al*, 1998). Resveratrol is a phyto-oestrogen, binding to and activating the oestrogen receptors that regulate the transcription of oestrogen-responsive target genes (Gehm *et al*, 1997) by either binding directly to DNA, at oestrogen response element (EREs), or by interacting with other transcription factors, for example, Sp1 (Sun *et al*, 1998), bound to their cognate sites on DNA (Bowers *et al*, 2000). Others have shown that steady-state *BRCA1* mRNA levels are elevated in response to oestrogens in human breast cancer cells, and that BRCA2 expression is also regulated by oestrogens in human breast cancer cell lines (Gudas *et al*, 1995; Spillman and Bowcock, 1996).

*BRCA1* and *BRCA2* are breast cancer susceptibility genes: inheritance of one defective copy of either of the two genes predisposes individuals to breast, ovarian and other cancers. The contribution of these genes to the pathogenesis of breast cancer is still unclear. No sporadic breast tumours have been shown to harbour mutations in the coding sequence of *BRCA1* or *BRCA2* (Miki *et al*, 1994; Foster *et al*, 1996). In contrast to normal breast epithelial cells, *BRCA1* mRNA levels in tumours appeared to be downregulated by methylation (Dobrovic and Simpfendorfer, 1997), while *BRCA2* showed significant overexpression in sporadic breast cancers (Bieche and Lidereau, 1999).

Here, we studied the effects of resveratrol on the expression of *BRCA1* and *BRCA2* in human breast cancer cell lines at the transcription level using quantitative real-time reverse transcription (RT)–PCR, and at the translation level by perfusion chromatography of the proteins.

## MATERIALS AND METHODS

### Cell cultures

MCF7 (Soule *et al*, 1973), MDA-MB 231 (Cailleau *et al*, 1974) and HBL 100 (Ziche and Gullino, 1982) cell lines were purchased from the ATCC (American Type Culture Collection, Manassas, VA, USA). Cells were cultured, respectively, in RPMI 1640, Leibovitz's L15 and McCoy's 5a medium (Life Technologies, Gaithersburg, MD, USA) supplemented with 2 mM L-glutamine, 20 *μ*g ml^−1^ gentamycin and 10% heat-inactivated FBS. Cells were grown in a humidified incubator with 5% carbon dioxide (except for MDA-MB 231 without CO_2_) at 37°C. Insulin (0.04 U ml^−1^) was added for MCF7 culture medium.

The ER status was checked in cell lines by immunocytochemistry with Centre Jean Perrin's anatomopathologist. MCF7 were found ER *α*+/*β*+, HBL 100 ER *α*−/*β*+ and MDA-MB-231 ER *α*−/*β*+.

### Resveratrol treatment of cells and flow cytometry analysis

MCF7, MDA-MB 231 and HBL 100 cells were maintained in medium supplemented with 10, 30 or 50 *μ*m *trans*-resveratrol (Sigma Chimie, St Quentin Fallavier, France) in DMSO for treated cells. A cell control was performed with DMSO. Cells were collected after 24, 48 or 72 h by trypsinisation and the DNA content was assessed by flow cytometry according to Krishan's (1975) method. Each experiment was performed in triplicate.

### Radiolabelling of cellular proteins

For cellular protein labelling, the cells were fed with 5 ml of medium supplemented with 100 *μ*Ci [^35^S] methionine (1000 Ci mM^−1^; Amersham International, Bucks, UK) and incubated for 20 h at 37°C in a 5% CO_2_ atmosphere. Metabolic radiolabelling was stopped by adding 10 ml cold PBS and cells were gently washed twice with PBS at 4°C. Labelled cells were solubilised in 750 *μ*l of 0.1 M Tris-HCl pH 7.1 containing 0.5% Nonidet P40 (NP 40; Boehringer Mannheim, Germany), sonicated for 2 min in ice and incubated at 4°C for 30 min. The insoluble material was removed by ultracentrifugation at 30 000 g for 30 min.

### Purification of DNA-binding proteins by affinity chromatography

The NP 40 cell lysates were loaded onto a POROS 20 HE (heparin) media column (PerSeptive Biosystems, Framingham, MA, USA). Proteins specifically bound to the gel were eluted with a gradient of NaCl from 0.1 to 1 M in 20 mM MES pH 5.5. The flow rate was 5 ml min^−1^ with a BioCAD Sprint high-performance liquid chromatography system (PerSeptive Biosystems, Framingham, MA, USA) equipped with a fraction collector (Gilson, Middleton, WI, USA). Fractions (0.5 ml) containing DNA-binding proteins were collected and pooled. Radioactivity was measured by adding 10 *μ*l of the collected fractions to 5 ml of scintillation cocktail (Packard Ready Safe) and counting.

### Isolation of *BRCA1* and *BRCA2* by affinity chromatography

BRCA1 or BRCA2 were immunoprecipitated from the previous eluate by addition of 16 *μ*g anti-BRCA1 polyclonal antibodies (556445; GeneTex, San Antonio, TX, USA) or anti-BRCA2 polyclonal antibodies (C-19; Santa Cruz Biotechnology) with a 30-min incubation at 37°C. The immunoprecipitate was isolated after fixation on POROS A column (PerSeptive Biosystems, Framingham, MA, USA) containing Protein A media and eluted 12 mM HCl/0.15 M NaCl, pH 2. Radioactivity of each 1 ml fraction was measured as described previously.

Immune complex elution from the Protein A column gave the amount of DNA-binding proteins that bound to anti-BRCA1 or anti-BRCA2 polyclonal antibodies, and a ratio was calculated as follows: 100 × (d.p.m. of BRCA1 or BRCA2 eluted from Protein A/d.p.m. of total DNA-binding proteins eluted from the heparin column). All data were expressed as means±s.d. of three assays (Hizel *et al*, 1999; Vissac *et al*, 2001).

### RNA extraction and cDNA synthesis

Total RNA was isolated using TRIZOL® (Gibco BRL, Carlsbad, CA, USA) according to the manufacturer's protocol. Total RNA (1 *μ*g) was used for the synthesis of first strand cDNA using the First Strand cDNA Synthesis kit (Amersham Pharmacia Biotech, Uppsala, Sweden) following the manufacturer's instructions.

### Determination of *BRCA1* and *BRCA2* mRNA using real-time quantitative RT–PCR

For *BRCA1* and *BRCA2* expression analysis, probes and primers were designed so that they overlapped splice junction, thereby avoiding the potential amplification of genomic DNA. The sequence of forward primers, TaqMan® probes and reverse primers were, respectively, for *BRCA1-exons (ex) 23/24* amplification: 5′-^5566^CAGAGGACAATGGCTTCCATG^5586^-3′, 5′-^5588^AATTGGGCA GATGTGTGAGGCACCTG^5613^-3′, 5′-^5646^CTACACTGTCCAACACCCACTCTC^5623^-3′; for *BRCA1-*ex 11/12 amplification: 5′-^4157^AAGAGGAACGGGCTTGGAA^4175^-3′, 5′-^4177^AAAATAATCAAGAAGAGCAAAGCATGGATTCAAACTTA^4214^-3′, 5′-^4236^CACACCCAGATGCTGCTTCA^4217^-3′; for *BRCA2-ex* 26/27 amplification: 5′-^9794^CCAAGTGGTCCACCCCAAC^9812^-3′, 5′-^9818^ACTGTACTTCAGGGCCGTACACTGCTCAAA^9847^-3′, 5′-^9895^CACAATTAGGAGAAGACATCAGAAGC^9870^-3′; for *BRCA2-ex* 12/13 amplification: 5′-^7120^GAAAATCAAGAAAAATCCTTAAAGGCT^7147^-3′, 5′-^7153^AGCACTCCAGATGGCACAATAAAAGATCGAAG^7184^-3′,5′-^7220^GTAATCGGCTCTAAAGAAACATGATG^7195^-3′. All doubly labelled probes, 18 S rRNA probe, primers plus TaqMan universal PCR master Mix were obtained from Applied Biosystems.

Multiplex PCR was carried out in 96-well plates on cDNA. A typical 25 *μ*l reaction sample contained 12.5 *μ*l TaqMan universel PCR Master Mix (dATP, dCTP, dGTP and dUTP, MgCl_2_, AmpliTaqGold, Amperase uracil-*N*-glycolsylase (UNG)), 200 nM of chosen *BRCA1* or *BRCA2* primers and TaqMan® probes, 50 nM of 18S rRNA primers and TaqMan® probe. Thermal cycling conditions were 2 min at 50°C and 10 min at 95°C followed with 40 cycles at 95°C for 15 s and 60°C for 1 min. Data were collected using the ABI PRISM 7700 SDS analytical thermal cycler (Applied Biosystems, Foster City, CA, USA).

Relative gene expression was determined using the comparative *C*_T_ (threshold cycle) method, which consists of the normalisation of the number of target gene copies to an endogenous reference gene (18S rRNA), designated as the calibrator (Fink *et al*, 1998). The level of *BRCA1*-ex 23/24, *BRCA1*-ex 11/12, *BRCA2*-ex 12/13 or *BRCA2*-ex 26/27 mRNA expression in each treated cell line was then normalised to the result obtained in the untreated cells. The amount of target, normalised to the 18S rRNA endogenous reference is given by the formula: 2^−ΔΔC_T_^. To guarantee the reproducibility of mRNA determination, two independent total RNA extractions were performed. Two independent RTs were carried out for one RNA extraction, while only one was performed for the second extraction. Each RT was analysed in triplicate and expressed as a mean±s.d. (Favy *et al*, 2000).

## RESULTS

### Cell proliferation by DNA content analysis

Treatments with 10, 30 and 50 *μ*M resveratrol were studied by flow cytometry after different times of exposure (24, 48 and 72 h) in MCF7, MDA-MB 231 and HBL 100 breast tumour cell lines. After exposure, all three cell lines were blocked in S phase. At 48 h, the percentage of cells in S phase was considerably increased after treatment with 30 *μ*M resveratrol, whereas the percentage of cells in G1 phase was decreased. It is well known that resveratrol treatment causes an accumulation of cells in S phase (Ragione *et al*, 1998).

### Analysis of the impact of resveratrol on *BRCA1* and *BRCA2* mRNA level

We compared the levels of *BRCA1* mRNA after treatment with 30 *μ*M resveratrol using two different *BRCA1* Taqman probes. The probe, *BRCA1-*ex 23/24, was used to quantify all *BRCA1* mRNA species together because no alternative splicing of ex 23 has been described (Wilson *et al*, 1997). The *BRCA1-*ex 11/12 probe was used to estimate the level of mRNA containing ex 11, because Thakur *et al* (1997) described the isolation and expression of two *BRCA1* cDNAs, one of them is a splicing variant generated by exclusion of ex 11 and producing a 4.6 kb mRNA. They observed a complex tissue-specific pattern of multiple spliced forms of *BRCA1*, and suggested that splicing may play a role in the regulation of BRCA1 function.

To evaluate *BRCA2* expression, we designed a TaqMan probe bridging exs 26–27, *BRCA2-*ex 26/27, because no alternative splicing has been observed so far, at this site. The *BRCA2-*ex 12/13 probe was used to estimate the level of mRNA containing ex 12, because Bièche and Lidereau (1999) identified an alternatively spliced *BRCA2* transcript that was widely expressed in all normal tissues examined. This Δ12-*BRCA2* transcript was found to be overexpressed in steroid receptor-negative breast tumour tissues, suggesting that dysregulation of the Δ12-*BRCA2* isoform may contribute to progression in human breast cancer.

**Figure 1:**
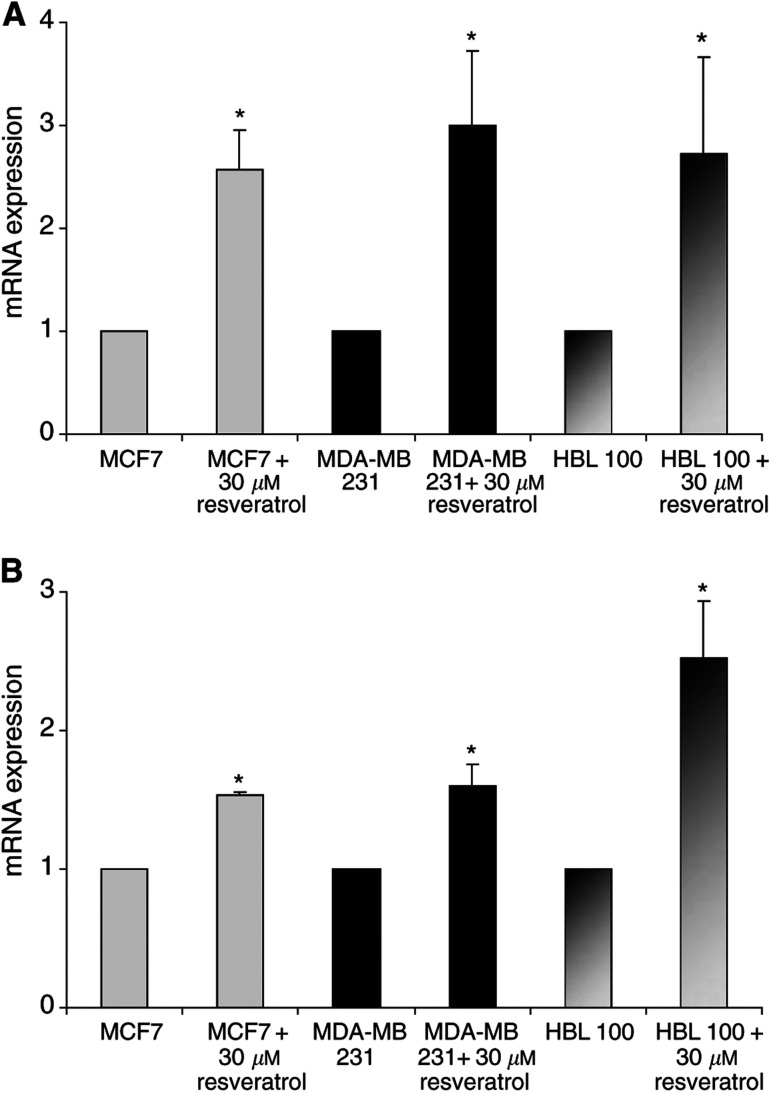
Effect of resveratrol on *BRCA1*-ex 23/24 (**A**) and *BRCA1*-ex 11/12 (**B**) mRNA levels in MCF7, MDA-MB 231 and HBL 100 after 48 h of treatment. Expression in treated cells was normalised to untreated controls (corresponding to arbitrary value: 1). Each measure was performed on two extractions and three RT and is expressed as mean±s.d. Statistic analysis was performed using the Student's *t*-test (^*^: *P*<0.05).

Expression of *BRCA1* and *BRCA2* mRNA in resveratrol-treated cells was normalised to their expression levels in untreated cells, normalised to 1. As shown in Figure 1

, the expression of each *BRCA1* mRNA species (BRCA1-ex 23/24 and BRCA1-ex 11/12) was increased in all three cell lines after treatment.

**Figure 2:**
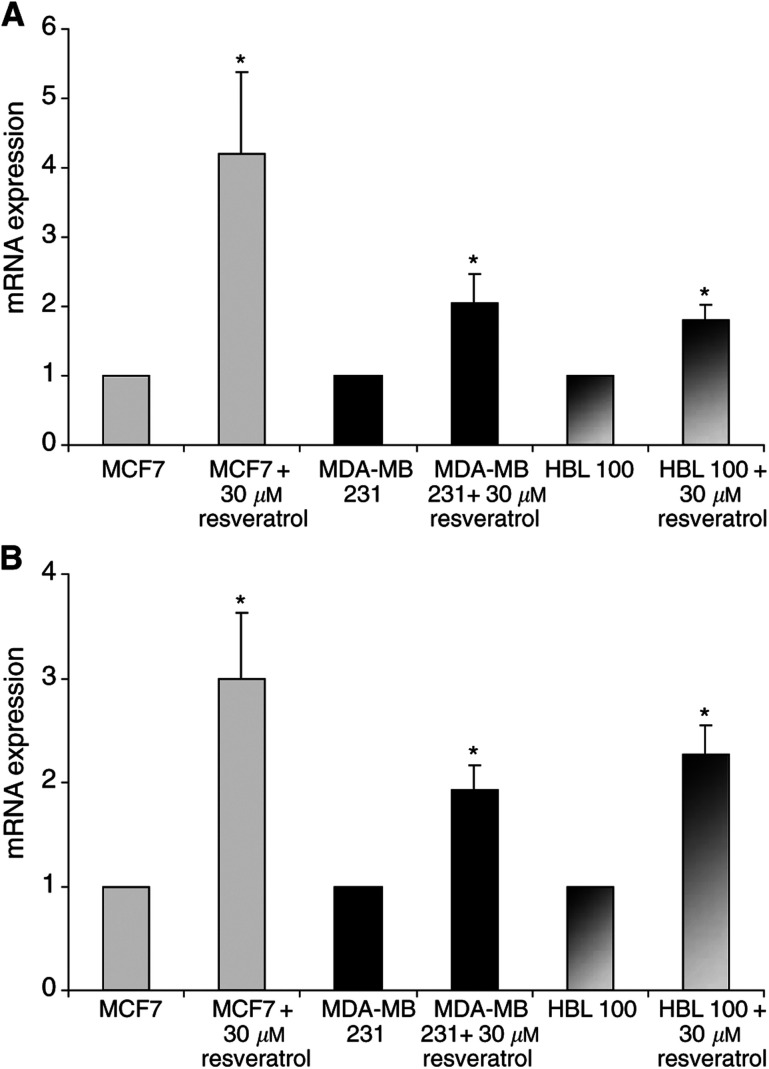
Effect of resveratrol on *BRCA2*-ex 26/27 (**A**) and *BRCA2*-ex 12/13 (**B**) mRNA levels in MCF7, MDA-MB 231 and HBL 100 after 48 h of treatment. Expression in treated cells was normalised to untreated controls (corresponding to arbitrary value: 1). Each measure was performed on two extractions and three RT and is expressed as mean±s.d. Statistic analysis was performed using the Student's *t*-test (^*^: *P*<0.05).

We also observed an increase of BRCA2-ex 26/27 and BRCA2-ex 12/13 mRNA in all three cell lines (Figure 2

). All mRNA determinations with treated cells were expressed as mean±s.d., with a student's *t*-test.

### Analysis of the impact of resveratrol on *BRCA1* and *BRCA2* protein synthesis

The amount of BRCA1 or BRCA2 protein was expressed as the following ratio: d.p.m. of labelled DNA-binding proteins bound specifically by the antibodies to BRCA1 or BRCA2/d.p.m. of total DNA-binding proteins purified by heparin chromatography (Table 1

**Table 1:** Amount of BRCA1 and BRCA2 proteins expressed by MCF-7, MDA-MB 231 and HBL 100 cells after treatment with 30 *μ*M resveratrol and 48 h exposure.

		Proteins assayed (%)	
	Cell lines	BRCA1	BRCA2
MCF-7	Untreated controls	0.58±0.01	1.00±0.03
	30 *μ*M resveratrol	0.51±0.02^*^	0.80±0.16^*^
MDA-MB231	Untreated controls	1.85±0.34	0.48±0.12
	30 *μ*M resveratrol	1.80±0.32	0.41±0.02
HBL100	Untreated controls	0.95±0.04	0.90±0.09
	30 *μ*M resveratrol	1.15±0.18	1.00±0.03

BRCA1 and BRCA2 proteins were obtained by DNA-binding protein purification, specific immunoprecipitation with anti-BRCA1 (556445) or anti-BRCA2 (C-19) antibodies and protein A affinity chromatography and expressed in percentage calculated as follows: 100 × d.p.m. DNA-binding proteins bound specifically to antibodies raised against BRCA1 or BRCA2/d.p.m. of total DNA-binding proteins purified on heparin affinity chromatography. All data are expressed as mean±s.e. of three assays (^*^), *P*<0.05, *vs* untreated controls (Student's *t*-test).

). BRCA1 and BRCA2 protein expression was not modified 48 h after treatment with 30 *μ*M resveratrol.

## DISCUSSION

We studied the effect of resveratrol, a natural polyphenolic compound found especially in black grapes, peanuts, berries and Itadori tea (Burns *et al*, 2002), on the expression of the *BRCA1* and *BRCA2* genes in the human breast cancer cell lines MCF7, MDA-MB 231 and HBL 100. We chose 48 h exposure to 30 *μ*M resveratrol because this treatment was shown to increase significantly the number of cells blocked in S phase (Park *et al*, 2001). It is well known that BRCA1 and BRCA2 reach their maximal levels in late G1 and S phases in normal and tumour-derived breast epithelial cells (Vaughn *et al*, 1996; Bertwistle *et al*, 1997).

Moreover, the effects of 30 *μ*M resveratrol correlated with results from others also studying resveratrol in cell lines (Ragione *et al*, 1998; Hsieh *et al*, 1999; Igura *et al*, 2001). In addition, we used a lower dose of 10 *μ*M resveratrol in the three cell lines (data not shown), but that did not show any accumulation of cells in S phase and consequently no significant alteration was found for *BRCA1, BRCA2* mRNA and BRCA1, BRCA2 proteins.

Then, the quantification of *BRCA1* and *BRCA2* mRNA was performed with real-time quantitative RT–PCR. This method allowed us to compare the effect of resveratrol by comparison with untreated cells, which were normalised to one. In MCF7, *BRCA1* mRNA increased 2.5-fold and *BRCA2* mRNA four-fold after 48 h in the presence of 30 *μ*M resveratrol. Similarly, in MDA-MB 231, *BRCA1* mRNA increased three-fold and *BRCA2* mRNA two-fold while in HBL 100, *BRCA1* mRNA increased 2.6-fold and *BRCA2* mRNA 1.9-fold.

The effect of resveratrol on *BRCA1* and *BRCA2* mRNA in human breast cancer cell lines could be explained by its different properties. First, it is structurally similar to the synthetic oestrogen, diethylstilbestrol, which exhibits oestrogenic activity. Gehm *et al* (1997) reported that resveratrol inhibited the binding of labelled oestradiol to the oestrogen receptor and activated transcription of oestrogen-responsive reporter genes transfected into human breast cancer cells. This transcriptional activation was oestrogen receptor-dependent, required an ERE in the reporter gene, and was inhibited by specific oestrogen antagonists. Moreover, resveratrol showed oestrogen agonist activity in MCF7 cells by activating the expression of two oestrogen-responsive genes, such as *progesterone receptor (PR)* and *pS2* genes (Jang and Pezzuto, 1999). And, we also found elsewhere an increase in *pS2* mRNA in MCF7, HBL100 and MDA-MB-231 human breast cancer cell lines (unpublished data). Resveratrol binds ER *β* and ER *α* with comparable affinity. However, resveratrol-liganded ER *β* has higher transcriptional activity than 17*β*-oestradiol-liganded ER *β*. This indicates that the cells that uniquely express ER *β* or that express higher levels of ER *β* than ER *α* may be more sensitive to resveratrol's oestrogen agonist activity (Bowers *et al*, 2000). Furthermore, the oestrogen agonist activity of resveratrol depends on the ERE sequence and the type of ER. Thus, in some cell types (e.g., MCF7 cells), resveratrol functioned as a superagonist (i.e., produced a greater maximal transcriptional response than oestradiol), whereas in others it produced activation equal to or less than that of oestradiol (Gehm *et al*, 1997). On the other hand, an ERE has been described in the promoter of *BRCA1* (Xu *et al*, 1997). This can explain the fold increases in *BRCA1* and *BRCA2* mRNA across the different cell types.

The steady-state levels of *BRCA1* and *BRCA2* mRNAs were shown to be coordinately elevated by oestrogen in human breast cancer cell lines MCF7 and BT 483 (Spillman and Bowcock, 1996). Elsewhere, the expression of *BRCA1* mRNA was induced from 2.5- to 5.0-fold by oestrogen in human breast cancer cell lines MCF7, and the BRCA1 protein was about three-fold (Romagnolo *et al*, 1998). In our work, we found a comparable increase, of two- to four-fold for *BRCA1* and *BRCA2* mRNA, by resveratrol in human breast cell lines MCF7, MDA-MB 231 and HBL 100. The ER status for MCF7 were found ER *α*+/*β*+, HBL 100 (ER *α*–/*β*+) and MDA-MB 231 (ER *α*–/ER *β*+).

BRCA1 and BRCA2 proteins were quantified using two successive perfusion affinity chromatographies. Resveratrol had no effect on the level of either BRCA1 or BRCA2. We displayed the specificity of the anti-BRCA1 and anti-BRCA2 polyclonal antibodies by competition with the synthetic peptides used to generate the antibodies. A complete displacement of the equilibrium was obtained in each case demonstrating the specificity of the antibodies (data not shown) (Hizel *et al*, 1999; Vissac *et al*, 2001, 2002).

We found an increase in *BRCA1* and *BRCA2* mRNA after treatment with resveratrol in breast cancer cell lines but no effect at the protein level. These result suggest an uncoupling between mRNA and protein levels under these conditions. A similar uncoupling of *BRCA1* mRNA and protein levels was detected in synchronised populations of immortalised MCF10 and 184B5 cells proliferation. In these two cell lines, *BRCA1* mRNA level was tightly regulated during the cell cycle while BRCA1 protein level remained constant. Thus, it has been shown that *BRCA1* mRNA is highly expressed in late G1 phase of the cell cycle, whereas conditions that lead to cell cycle exit downregulate the *BRCA1* mRNA (Gudas *et al*, 1995, 1996; Jin *et al*, 1997). There are several possible explanations for discrepancies between mRNA and protein level under different physiological conditions. *BRCA1* and *BRCA2* might be post-transcriptionally regulated with effects on the translational activity as well as the stability of *BRCA1* and *BRCA2* mRNA (Wickens *et al*, 1997). Alternatively, the level of *BRCA1* mRNA in cells may be translationally regulated by other cellular proteins or antisense RNA transcripts. Precedents for both mechanisms of regulation exist for other genes. Interestingly, many developmentally regulated genes exhibit regulation at the level of mRNA translation (Hentze, 1995). More recently, Blagosklonny *et al* (1999) demonstrated a substantial role for proteolysis in regulating BRCA1 steady-state protein levels in several cell lines. Degradation by a cathepsin-like protease in fine balance with *BRCA1* transcription is responsible for maintaining the low steady-state level of BRCA1 protein seen in many cancer cells. At the opposite of oestrogen, which increased the level of mRNAs and proteins of the two oncosuppressors *BRCA1* and *BRCA2*, the resveratrol seems to play a role in one of the different pathways of previous mechanisms.

To better understand the effects of resveratrol on *BRCA1* and *BRCA2* oncosuppressor genes in mammary gland, we will use cDNA microarrays to study gene-expression profiles of proteins interacting with BRCA1 and BRCA2 after phytochemical treatment and it would be helpful to study proteomics.

In conclusion, the present study demonstrates that 30 *μ*M resveratrol can increase expression of the *BRCA1* and *BRCA2* oncosuppressors, involved in the aggressiveness of human breast cancer cell lines.
